# Further evaluation of differential expression of keratoconus candidate genes in human corneas

**DOI:** 10.7717/peerj.9793

**Published:** 2020-08-20

**Authors:** Justyna A. Karolak, Barbara Ginter-Matuszewska, Katarzyna Tomela, Michal Kabza, Dorota M. Nowak-Malczewska, Malgorzata Rydzanicz, Piotr Polakowski, Jacek P. Szaflik, Marzena Gajecka

**Affiliations:** 1 Chair and Department of Genetics and Pharmaceutical Microbiology, Poznan University of Medical Sciences, Poznan, Poland; 2 Institute of Human Genetics, Polish Academy of Sciences, Poznan, Poland; 3 Department of Medical Genetics, Medical University of Warsaw, Warsaw, Poland; 4 Department of Ophthalmology, Medical University of Warsaw, Warsaw, Poland

**Keywords:** Gene expression, TGF-β pathway, Wnt signaling, Hippo signaling, CTGF, ZNF469, TGFB3, TGFBI, Keratoconus genetics, Keratoconic cornea

## Abstract

**Background:**

Keratoconus (KTCN) is a progressive eye disease, characterized by changes in the shape and thickness of the cornea that results in loss of visual acuity. While numerous KTCN candidate genes have been identified, the genetic etiology of the disease remains undetermined. To further investigate and verify the contribution of particular genetic factors to KTCN, we assessed 45 candidate genes previously indicated as involved in KTCN etiology based on transcriptomic and genomic data.

**Methods:**

The RealTime ready Custom Panel, covering 45 KTCN candidate genes and two reference transcripts, has been designed. Then, the expression profiles have been assessed using the RT-qPCR assay in six KTCN and six non-KTCN human corneas, obtained from individuals undergoing a penetrating keratoplasty procedure.

**Results:**

In total, 35 genes exhibiting differential expression between KTCN and non-KTCN corneas have been identified. Among these genes were ones linked to the extracellular matrix formation, including collagen synthesis or the TGF-β, Hippo, and Wnt signaling pathways. The most downregulated transcripts in KTCN corneas were *CTGF, TGFB3, ZNF469, COL5A2, SMAD7*, and *SPARC*, while *TGFBI* and *SLC4A11* were the most upregulated ones. Hierarchical clustering of expression profiles demonstrated almost clear separation between KTCN and non-KTCN corneas. The gene expression levels determined using RT-qPCR showed a strong correlation with previous RNA sequencing (RNA-Seq) results.

**Conclusions:**

A strong correlation between RT-qPCR and earlier RNA-Seq data confirms the possible involvement of genes from collagen synthesis and the TGF-β, Hippo, and Wnt signaling pathways in KTCN etiology. Our data also revealed altered expression of several genes, such as *LOX*, *SPARC*, and *ZNF469*, in which single nucleotide variants have been frequently identified in KTCN. These findings further highlight the heterogeneous nature of KTCN.

## Introduction

Keratoconus (KTCN) is a progressive eye disorder characterized by thinning and conically shaped protrusion of the cornea, resulting in severe visual impairment (Rabinowitz, 1998). The first symptoms of KTCN usually appear during the second or third decade of life (Rabinowitz, 1998). However, individuals with early-onset KTCN have also been reported (Léoni-Mesplié et al., 2012). The estimated prevalence of KTCN varies between 1 in 375 and 1 in 2,000 individuals and depends on geographic location and ethnic origin (Godefrooij et al., 2016).

Keratoconus is considered a multifactorial disease with a substantial genetic contribution, supported by evidence from twin, family, and population studies (Tuft et al., 2012; Naderan et al., 2016; Lu et al., 2013). Because of the complex etiology and the genetic heterogeneity of the disease, the identification of specific risk factors for KTCN is difficult. However, the use of various approaches has already allowed detection of many candidate genes that could be implicated in KTCN as have been previously reviewed (Nowak & Gajecka, 2011; Abu-Amero, Al-Muammar & Kondkar, 2014; Bykhovskaya, Margines & Rabinowitz, 2016; Karolak & Gajecka, 2017; Valgaeren, Koppen & Van Camp, 2018; Loukovitis et al., 2018; Lucas & Burdon, 2020).

More than 20 chromosomal regions linked to KTCN have been identified (Hameed et al., 2000; Fullerton et al., 2002; Tyynismaa et al., 2002; Hughes et al., 2003; Brancati et al., 2004; Hutchings et al., 2005; Tang et al., 2005; Li et al., 2006; Burdon et al., 2008; Bisceglia et al., 2009; Gajecka et al., 2009; Liskova et al., 2010; Rosenfeld et al., 2011; Nowak et al., 2013), including loci in 5q21.2 and 5q31.1-q35.3 which have been replicated independently in different populations (Tang et al., 2005; Li et al., 2006; Bisceglia et al., 2009; Rosenfeld et al., 2011; Bykhovskaya et al., 2016b). Linkage studies also have led to the identification of a few potential KTCN candidate genes, including *SPARC, IL1RN*, and *SKP1*. These genes encode a cysteine-rich acidic matrix-associated protein, IL1 receptor antagonist, and S-phase kinase-associated protein 1 that are known to be involved in the extracellular matrix (ECM) formation, immune response, and ubiquitination, respectively (Bisceglia et al., 2009; Nowak et al., 2013; Karolak et al., 2016a).

Genome-wide association studies (GWAS) have identified several loci that show significant or suggestive association with KTCN, including the promoter and upstream region of the hepatocyte growth factor (*HGF*) gene (Burdon et al., 2011; Sahebjada et al., 2014), as well as the lysyl oxidase (*LOX*) (Bykhovskaya et al., 2012; Hasanian-Langroudi et al., 2015), and zinc finger protein 469 (*ZNF469*) (Lu et al., 2013; Sahebjada et al., 2013) genes. Although the exact role of these genes in KTCN etiology is not fully established, some evidence suggested their involvement in ECM homeostasis in the human cornea (Burkitt Wright et al., 2011; Burdon et al., 2011; Dudakova et al., 2012).

A gene approach, utilizing a direct screening of functional candidate genes for KTCN, has allowed the detection of numerous promising genes implicated in KTCN etiology. Among them were visual system homeobox 1 (*VSX1*) and superoxide dismutase isoenzyme 1 (*SOD1*) (Héon et al., 2002; Udar et al., 2006), involved in wound healing (Barbaro et al., 2006) and oxidative stress (Udar et al., 2006), respectively. Other candidates genes include transforming growth factor beta-induced (*TGFBI*) or zinc-finger E-box binding homeobox 1 (*ZEB1*), involved in corneal dystrophies (Guan et al., 2012; Lechner et al., 2013) or *COL4A1, COL4A2, COL4A2*, and *COL4A1* (Stabuc-Silih et al., 2009; Karolak et al., 2011), encoding collagen proteins responsible for the proper function of the cornea.

Gene expression studies are also a valuable source of information about functional genes and pathways important for KTCN pathogenesis. A high-throughput RNA sequencing (RNA-Seq) showed a serious alteration of numerous genes in KTCN corneas (Kabza et al., 2017). The most significant downregulation was observed among genes belonging to the TGF-β, Hippo, and Wnt signaling and collagen synthesis and maturation networks that are responsible for proper corneal organization and regulation of corneal ECM remodeling (Kabza et al., 2017). Abnormalities within genes involved in ECM have also been reported in subsequent RNA- and DNA-based studies (Khaled et al., 2018; You et al., 2018; Sharif et al., 2019; Karolak et al., 2020). Thus, disruptions within these molecular cascades could be potentially responsible for corneal changes underlying the development of KTCN.

While the genes mentioned above have been hypothesized to play a role in KTCN, some of these identified factors account for a limited number of individuals only being found in single families or particular studied populations. Also, in some cases, the results of the initial findings are contradictory. Thus, in this study, we have taken advantage of the previous RNA- and DNA-based KTCN analyses to select 45 candidate genes for further investigation aiming to validate their contribution to KTCN. The possible involvement of these genes in disease etiology was re-evaluated using the RealTime ready Custom Panels in the RT-qPCR experiments, performed in KTCN and non-KTCN corneas.

## Methods

### Subjects

Six KTCN patients and six non-KTCN individuals were enrolled in this study and underwent a complete ophthalmic evaluation. The KTCN diagnosis was made based on the previously described criteria (Karolak et al., 2016b; Kabza et al., 2017). The clinical characteristics of all enrolled individuals are shown in Table S1. All individuals provided written informed consent for participation in the study, in accordance with the Declaration of Helsinki. The research protocol was approved by the Institutional Review Board at Poznan University of Medical Sciences (453/14 and 755/19).

### Material

The corneas were obtained from previously evaluated Polish KTCN (KC15, KC16, KC17, KC18, KC19, KC20) and non-KTCN (KR19, KR21, KR23, KR24, KR25, KR49) individuals, undergoing penetrating keratoplasty procedure (Karolak et al., 2016b; Kabza et al., 2017). Six non-KTCN corneas, used as controls, were collected from patients who were referred for corneal transplantation for different reasons, including corneal ulcer (KR21), bullous keratopathy (KR23, KR25), and history of the ocular trauma (KR24, KR49) (Table S1). RNA samples from all corneal tissues have already been extracted with a Total RNA Purification Kit (Norgen Biotek, Thorold, ON, Canada) as described (Kabza et al., 2017). All but one (KR49) RNA samples used in this study have also been assessed in the prior comprehensive RNA-Seq experiment (Kabza et al., 2017).

### Candidate gene assay design

Forty-five genes were selected for further evaluation based on both corneal RNA-Seq results and previous genomic data. The first KTCN candidate gene set included 26 genes that have been differentially expressed in our earlier studies performed in KTCN and non-KTCN corneas. These genes were selected from the top molecular pathways overrepresented across deregulated genes and encode core elements of collagen synthesis and maturation pathways, the TGF-β, Hippo, and Wnt signaling pathways, as well as their potential regulators (Kabza et al., 2017). The second set consists of 19 genes that have been previously reported as involved in KTCN based on the detection of putative variants within the gene, localization within the linkage region, or proximity to variants associated with KTCN. The selected genes are involved in inflammation, intracellular signaling, ubiquitination, and other processes reported as crucial for the functioning of the eye or disease development. The complete list of studied genes is presented in Table S2.

### RT-qPCR analysis

To analyze gene expression profiles, total RNA samples from six KTCN to six non-KTCN corneal tissues were reverse transcribed to cDNA with the Transcriptor First Strand cDNA Synthesis Kit (Roche Diagnostics, Penzberg, Germany), according to the manufacturer’s procedure. Expression levels of 45 candidate KTCN genes and two housekeeping transcripts (Table S2) were assessed using the RealTime ready Custom Panel 96 (Roche Diagnostics, Penzberg, Germany), containing pre-plated primer pairs and probes. RT-qPCR analyses were conducted using the LightCycler 96 System (Roche Diagnostics, Penzberg, Germany) in a total volume of 20 μl containing the FastStart Essential DNA Probe Master (Roche Diagnostics, Penzberg, Germany), and 0.5 ng of cDNA. Each reaction was performed in triplicate.

### RT-qPCR data analysis

Relative quantification of the gene expression was normalized to the level of the *GAPDH* and *IPO8* transcripts with the comparative C_T_ method. The R environment was used to analyze differential expression and to generate a heat map showing hierarchical clustering (Ward linkage) of KTCN and non-KTCN samples based on the expression profiles (R Development Core Team, 2008). The statistical significance of differential gene expression was evaluated using Student’s *t*-test. The adjusted *P*-values were calculated using the Benjamini–Hochberg (FDR) procedure.

### Comparison of RT-qPCR data with RNA-Seq data

The log2 transformed fold change (FC) values of gene expression levels between KTCN and non-KTCN samples were calculated for RT-qPCR and previously reported RNA-Seq data, GSE77938 (Kabza et al., 2017). To calculate the log2 FC values based on RNA-Seq results, gene expression data from both discovery and replication RNA-Seq experiments were analyzed together. Briefly, Illumina adapter sequences, poor quality regions (average Phred quality score below five), and sequences matching human rRNAs were removed from the sequenced short reads using the BBDuk2 program from the BBTools package (http://jgi.doe.gov/data-and-tools/bbtools). Filtered reads shorter than 50 bp were discarded. The expression values of known genes and transcripts from GENCODE 25 annotations (Ensembl 87) were estimated using Salmon with sequence–specific and GC content bias correction enabled. RNA–Seq FC values were obtained from *limma* package using a previously published protocol (Ritchie et al., 2015; Patro, Duggal & Kingsford, 2015; Law et al., 2016; Kabza et al., 2017). Pearson correlations between log2 FC values for selected genes obtained from RT-qPCR and RNA-Seq data were evaluated using the R package (R Development Core Team, 2008).

## Results

### Differential expression analysis

Nine of 45 studied genes (*BMP4, PPP2R2B, SMAD9, IL6, IL17B, PLEKHA7, FGF14, MORC1*, and *SYN2*) had high frequency (>8.5%) of missing data and were excluded from further analyses. In result, gene expression profiles were assessed in six KTCN and six non-KTCN corneas for 36 candidate KTCN genes (Table 1).

**Table 1:** Changes in the gene expression level determined using RT-qPCR in the KTCN corneas compared with the non-KTCN corneas.

Gene	*P* value_adjusted	FC	log2 FC
(A) Genes selected based on RNA-seq study from top molecular pathways overrepresented across deregulated genes, encoding core elements of collagen synthesis and maturation pathways, the TGF-β, Hippo, and Wnt signaling pathways, as well as their potential regulators			
*ACTB*	0.133475711	0.644242264	−0.6343248
*BMP1*	0.113652146	0.525332459	−0.9286974
*COL21A1*	0.590726465	1.655146746	0.7269591
*COL5A2*	0.128826872	0.183439285	−2.4466255
*CTGF*	0.113652146	0.055297401	−4.1766445
*DNMT1*	0.360409941	0.710043749	−0.4940202
*DNMT3A*	0.214127903	1.303679974	0.3825898
*DNMT3B*	0.219396173	0.543577449	−0.8794425
*EZH2*	0.184023747	0.741651443	−0.4311868
*LOX*	0.2178701	0.261069481	−1.9374943
*SMAD7*	0.076671938	0.217447449	−2.2012613
*TEAD2*	0.113652146	0.262175317	−1.9313962
*TEAD3*	0.321839445	1.255782411	0.3285865
*TEAD4*	0.1055257	0.357861338	−1.4825274
*TGFB1*	0.113652146	0.443800498	−1.1720168
*TGFB2*	0.405724701	0.571357569	−0.8075342
*TGFB3*	0.076671938	0.165798806	−2.5924945
*TGFBR1*	0.199938575	0.760414605	−0.3951419
*TGFBR2*	0.113652146	0.480308326	−1.0579673
*WNT5A*	0.710742768	0.904652905	−0.1445637
*YY1*	0.751277265	1.092918686	0.1281861
*YY1AP1*	0.367206418	1.193550658	0.2552598
*ZFYVE9*	0.314142017	1.400497193	0.4859391
(B) Genes previously reported as involved in KTCN based on their function, detection of putative variants within the gene, localization within the linkage region, or localization in proximity to variants associated with KTCN			
*CTNNB1*	0.994110913	1.000820444	0.0011832
*DOCK9*	0.425606848	1.313649696	0.3935806
*FGF9*	0.556166983	1.531641011	0.6150782
*HGF*	0.826503411	0.870795372	−0.1995944
*IL1RN*	0.271724961	0.791829931	−0.3367375
*PROB1*	0.271724961	0.628154772	−0.6708080
*SKP1*	0.077097944	1.632464428	0.7070516
*SLC4A11*	0.133475711	2.408981021	1.2684230
*SPARC*	0.128826872	0.226367696	−2.1432600
*TGFBI*	0.113652146	2.488055868	1.3150189
*WDR33*	0.292948374	1.306800874	0.3860393
*ZEB1*	0.321839445	0.469715758	−1.0901401
*ZNF469*	0.076671938	0.091553859	−3.4492355

The analysis of RT-qPCR data showed that 23 genes had decreased expression level in the KTCN patients compared to the non-KTCN individuals, including six genes (*CTGF, ZNF469, TGFB3, COL5A2, SMAD7*, and *SPARC*) with log2 FC values lower than −2.0 and six genes (*LOX, TEAD2, TEAD4, TGFB1, TGFBR2*, and *ZEB1*) with log2 FC values between −1.0 and −1.99 (Table 1). Twelve genes presented with increased expression, including *SLC4A11* and *TGFBI* with log2 FC values greater than 1.0 (Table 1). No difference between the level of *CTNNB1* transcripts was observed for KTCN and non-KTCN individuals. Relative gene expression is presented in Table S3.

**Figure 1:**
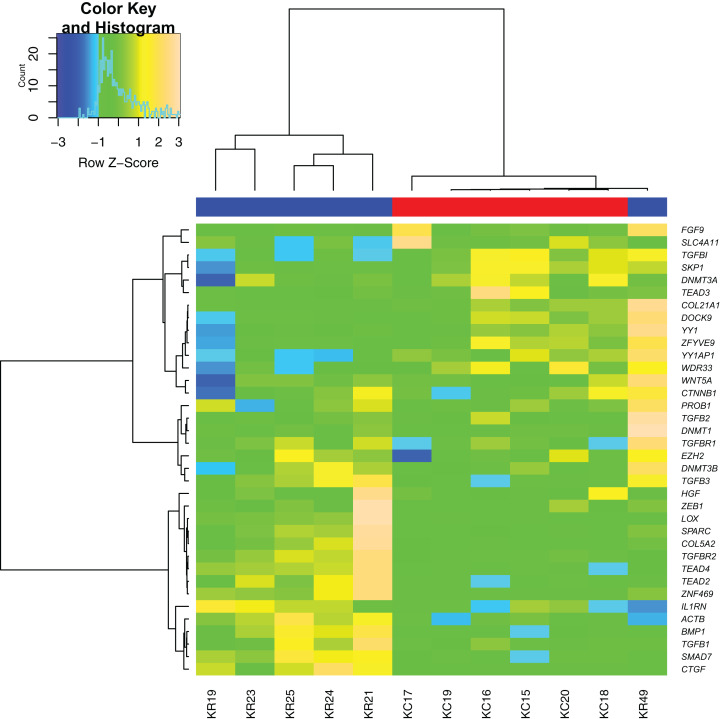
Hierarchical clustering analysis of gene expression. A heat map indicates hierarchical clustering (Ward linkage) of keratoconus (KTCN) and non-KTCN samples (KR) based on the expression values of 36 analyzed genes. Data was clustered as groups of genes (vertical line) and groups of individuals (horizontal line). Color scale encodes *Z*-score of gene expression; results indicated in green and red point to expression values above and below the median, respectively. Upper color labeling shows KTCN samples in red and non-KTCN samples in blue.

Sample clustering performed based on all differentially expressed genes showed almost clear separation of KTCN and non-KTCN clusters. Only one non-KTCN sample (KR49) was misclassified in the KTCN cluster (Fig. 1).

### Comparison of RT-qPCR data with RNA-seq data

**Figure 2:**
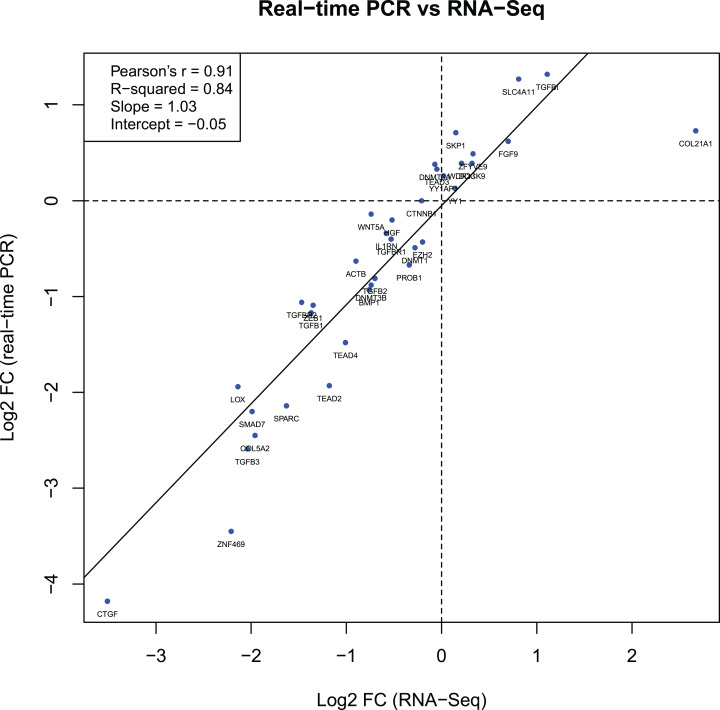
Gene expression correlations between RT-qPCR and RNA-Seq data. The diagram shows the relationship between binary logarithms (log2) of the fold change values obtained from RT-qPCR (vertical axis) and RNA-Seq (horizontal axis) experiments for 36 analyzed genes. The Pearson correlation and linear model coefficients, as well as linear regression line are indicated.

The log2 FC values for 36 genes obtained during the RT-qPCR and RNA-Seq showed a strong correlation between the two sets of results. The Pearson correlation coefficient (*r*) was 0.91 (Fig. 2).

## Discussion

Differences in transcriptomic profiles between various cells and tissues play an essential role in the normal functioning of the human body (Frith, Pheasant & Mattick, 2005; Cummings et al., 2017). During the last few years, numerous transcriptome studies have been performed to understand how the changes in gene expression may influence different phenotypes, including KTCN (Nielsen et al., 2003; Macé et al., 2011; Joseph, Srivastava & Pfister, 2016; Kabza et al., 2017).

The results of RNA-Seq-based transcriptomic profiling of human corneas suggested that downregulation of collagens and other genes encoding ECM components might be involved in KTCN etiology as it can lead to the reduction of the mechanical stability of affected tissues (Kabza et al., 2017). Moreover, it was postulated that the previously observed decreased level of collagen genes in KTCN corneas might be a result of downregulation of connective tissue growth factor (*CTGF*) and its modulators, which are involved in the TGF-β, Hippo, and Wnt signaling pathways (Kabza et al., 2017). Interestingly, changed expression patterns of the core elements of these three biological pathways in KTCN corneas were confirmed in the present RT-qPCR study.

Three transforming growth factor beta (TGF-β) isoforms, TGFB1, TGFB2, and TGFB3, through binding to their receptors (TGFBR1, TGFBR2), initiate signaling that regulates the assembly of ECM. While TGFB1 and TGFB2 act as stimulators of a profibrotic response in the injured cornea, TGFB3 is known for its antifibrotic effect (Priyadarsini et al., 2015). TGF-β signaling stimulates the downstream synthesis of CTGF. As a result, corneal cells are activated and begin to produce different types of collagen and other ECM components (Blalock et al., 2003). The levels of *TGFB1*, *TGFB3*, *TGFBR2*, *COL5A2*, and *CTGF* were decreased in this study confirming that previously observed low expression of genes encoding major ECM components, including collagens, may be an effect of TGF-β signaling alteration in KTCN (Joseph, Srivastava & Pfister, 2016; Kabza et al., 2017).

The TGF-β pathway is controlled by SMAD proteins, including SMAD family member 7, which through binding to TGF-β receptors, leads to an inhibition in TGF-β signaling (Yan, Liu & Chen, 2009). Interestingly, TGF-β transcriptional responses could also be regulated by non-Smad pathways (Zhang, 2009). That could explain decreased SMAD7 level and increased expression of TGFBI, a downstream effector of TGF-β, at the same time. The TGFBI is a significant component of human corneal stroma involved in cell adhesion and migration, which is induced by its interaction with several ECM elements, including collagens (Runager, Enghild & Klintworth, 2008). *TGFBI* variants have been frequently identified in patients with corneal dystrophies (Chao-Shern et al., 2019), as well as KTCN (Guan et al., 2012). While a significant upregulation of TGFBI in KTCN patients compared to control individuals was also detected previously (Bykhovskaya et al., 2016a), in most transcriptomic or proteomic studies the level of TGFBI in KTCN tissues was decreased (Takács et al., 1999; Zhao et al., 2002). Since different material was used in these mentioned analyses (whole corneas, corneal buttons, or corneal stroma), the observed results could vary because of the influence of other cells on the tissue expression. More studies are needed to verify this data.

It is known that TGF-β is integrated into higher-order networks and crosstalk between TGF-β, Wnt and Hippo signaling regulates the outcome of signaling activity (Attisano & Wrana, 2013). The Hippo molecular cascade is involved in controlling the eye size, and TEA domain transcription factors, encoded by the *TEAD2* and *TEAD4* genes, play an essential role in its regulation (Yu & Guan, 2013). Moreover, TEAD2 and TEAD4 transcription factors integrate Hippo and Wnt pathways at the nucleus through cooperation with YAP and TAZ molecules (Attisano & Wrana, 2013). Decreased level of *TEAD2* and *TEAD4*, observed in this study in KTCN corneas, supports our previous data. Also, it indicates that abnormal expression of particular elements of the TGF-β, Hippo, and Wnt pathways might alter signaling crosstalk between these cascades in KTCN. Of note, our recent ES findings showed the accumulation of variants in several genes from Wnt signaling and/or focal adhesion pathways, deregulated in KTCN, further supporting possible involvement of these genes in disease etiology (Karolak et al., 2020).

Apart from genes involved in the pathways mentioned above, selected as candidates for KTCN based on solely transcriptome study, there are numerous putative KTCN genes, which are revealed using linkage analyses, GWAS, or ES (Karolak & Gajecka, 2017). The level of these genes expression was also evaluated using RNA-Seq (Kabza et al., 2017). However, their significance in KTCN could not be clearly defined due to contradictory findings in genomic studies. Thus, in this study, 19 candidate KTCN genes were selected for re-evaluation using RT-qPCR. In KTCN corneas compared to non-KTCN corneas, we observed altered expression profiles of a few of them, including *ZNF469, SPARC*, and *LOX*.

The *ZNF469* gene was initially associated with central corneal thickness, which is abnormal in KTCN or corneal dystrophies (Lu et al., 2010, 2013; Vitart et al., 2010) and now is one of the most discussed KTCN genes. Another KTCN candidate, the *SPARC* gene, is positioned in the 5q32-q33 region that shows a suggestive linkage with KTCN in previous studies (Bisceglia et al., 2009). A lot of single nucleotide variants in *ZNF469* (Vincent et al., 2014; Lechner et al., 2014; Davidson et al., 2015; Karolak et al., 2016b; Kalantan et al., 2017; Lucas et al., 2017) and *SPARC* (De Bonis et al., 2011) have been identified in KTCN patients. However, the role of both genes in determining the disease has not been definitively clarified based on mutational studies and it should be further evaluated in the KTCN context. The proteins encoded by both *ZNF469* and *SPARC* regulate ECM production and remodeling and participate in collagen homeostasis in the human cornea (Bradshaw et al., 2003; Burkitt Wright et al., 2011). Thus, the observed decreased expression of both genes in KTCN corneas might also partially explain previously identified downregulation of genes encoding ECM components and might shed more light on the role of these genes in KTCN.

The *LOX* gene encodes an enzyme responsible for collagen cross-linking in the cornea and is one of the well-studied genes in KTCN. It is mapped within 5q21 region linked to KTCN in Italian population (Bisceglia et al., 2009). While the first mutational screening of *LOX* has not confirmed the involvement of *LOX* variants in KTCN, evidence of a genetic association between KTCN and common SNVs located in the *LOX* gene has been revealed in Caucasian (Bykhovskaya et al., 2012), Czech (Dudakova et al., 2015), Iranian (Hasanian-Langroudi et al., 2015), Brazilian (Gadelha et al., 2020), and Han Chinese (Xu et al., 2020) KTCN patients. In our study, the level of *LOX* was decreased in KTCN corneas. Previously, the reduced expression or activity of *LOX* have been found in the cultured keratoconic fibroblasts (Dudakova et al., 2012), KTCN corneal epithelium and tears (Shetty et al., 2015), the cone apex of KTCN patients (Pahuja et al., 2016), as well as in the whole KTCN corneas (Kabza et al., 2017). The observed decreased level of *LOX* might affect the reduction of collagen cross-linking in the corneal stroma, leading to the thinning of the cornea (Dudakova et al., 2012; Pahuja et al., 2016).

Among the genes with detected increased expression in KTCN, the most interesting candidates are *TGFBI*, discussed above, and *SLC4A11*. Sodium bicarbonate transporter-like protein 11 (SLC4A11), functioning as an electrogenic Na^+^-coupled borate co-transporter, is involved in the stimulation of cell growth and proliferation via borate-dependent mitogen-activated protein kinase activation (Romero, Fulton & Boron, 2004; Park et al., 2004). *SLC4A11* has been reported as involved in KTCN and its observed deregulation supports previous findings (Guan et al., 2012; Nowak et al., 2013). However, the mechanism through which they influence KTCN development is unknown and further research should be performed to interpret the obtained data.

In our study, hierarchical cluster analyses of RT-qPCR gene expression data correctly grouped the majority of KTCN and non-KTCN corneas. However, one non-KTCN sample (KR49) was misclassified with the KTCN cluster. As was hypothesized previously, the observed misclassification might be the consequence of the high heterogeneity of the control group, which could be recognized as a study limitation (Kabza et al., 2017). As it was not possible to obtain “healthy” corneas from living donors, corneal tissues have been ascertained from control individuals with different ocular phenotypes, including corneal ulcer, bullous keratopathy, and history of keratitis and ocular trauma. All of these diseases can result in corneal scarring. The misclassified KR49 cornea was obtained from a 38-years-old man referred for corneal transplantation after ocular trauma and had no other eye diseases. The tissue homeostasis during corneal scarring could be altered due to activation or inhibition of several molecular pathways. Depending on the stage of wound healing that results in ocular scarring, the gene expression of particular genes could be abnormal. Thus, we hypothesize that this could lead to the misclassification of our patient in hierarchical cluster analyses based on gene expression profiles. In addition, KR49 patient was the youngest individual among control participants, which could also be a potential confounding factor.

Another aspect of our study was to validate the RNA-Seq experiment performed previously in the same (except for KR49) human KTCN and non-KTCN corneas. The comparison of RNA-Seq data with results obtained from current RT-qPCR analysis showed a strong correlation between the two sets of data, indicating that the selected 36 genes were not false positives and suggesting high concordance between both methods. However, protein-based assays would provide further validity to observed findings.

## Conclusions

In conclusion, in the present study, we have assessed the expression of 36 candidate genes across KTCN and non-KTCN human corneas to further investigate their contribution in KTCN etiology. We confirmed differential expression of 35 genes, including core elements of recently postulated KTCN pathways: the ECM formation, Hippo, and Wnt signaling. We found that the deregulation of genes identified using RNA-Seq was not incidental, and selected candidate genes were not false positives. Our results also showed abnormal expression of several other genes, such as *LOX*, *SPARC*, *ZNF469*, or *TGFBI*, in which single nucleotide variants have been frequently identified in KTCN individuals. Although the obtained results partially explain the molecular basis of KTCN etiology, our understanding of this complex disease is still rudimentary. To address this gap, further studies in a larger group of patients should be performed.

## Supplemental Information

10.7717/peerj.9793/supp-1 Supplemental Information 1 The clinical characteristics and ophthalmic findings of the examined individuals, keratoconus (KC) and non-keratoconus (KR) subjects. Abbreviations in table: OD – right eye, OS – left eye, OU – both eye, nd – data not available, IOP - intraocular pressure, AL - axial length, DES - dry eye syndrome *italic font in AL [mm] column indicates a measurement made before surgery

10.7717/peerj.9793/supp-2 Supplemental Information 2 Genes selected for RT-qPCR analysis using Real Time ready Custom Panels (Roche).

10.7717/peerj.9793/supp-3 Supplemental Information 3 Relative changes in gene expression level (rsd %) in the KTCN corneas (KC) compared with the non-KTCN corneas (KR). Mean expression (mean) and standard deviations (sd) are shown.

10.7717/peerj.9793/supp-4 Supplemental Information 4 RT-qPCR data for each sample ( 2 ^-ΔΔCt) used for FC calculations.

## Additional Information and Declarations

### DNA Deposition

The following information was supplied regarding the deposition of DNA sequences: The RNA-seq data are available in the Gene Expression Omnibus database (GSE77938).

### Data Availability

The following information was supplied regarding data availability: The raw data are available in Table S4.
