# Supplementary material for: Further evaluation of differential expression of keratoconus candidate genes in human corneas
Source: PeerJ. 2020 Aug 20;8:e9793. doi: 10.7717/peerj.9793 (PMC7443321; doi:10.7717/peerj.9793)
Supplement: Supplemental Information 1 — Abbreviations in table: OD – right eye, OS – left eye, OU – both eye, nd – data not available, IOP - intraocular pressure, AL - axial length, DES - dry eye syndrome *italic font in AL [mm] column indicates a measurement made before surgery [file peerj-08-9793-s001.docx]

Table S1. The clinical characteristics and ophthalmic findings of the examined individuals, keratoconus (KC) and non-keratoconus (KR) subjects

| **Id** | **Status** | **Gender** | **Age at examination [year]** | **diff BFS [µm]** | | **Min. Pachymetry [µm]** | | **AL [mm]*** | | **IOP [mmHg]** | | **Eye rubbing** | **Using contact lens after KTCN diagnosis** | **Using contact lens before KTCN diagnosis** | **Ocular diseases** | **Other diseases** |
| --- | --- | --- | --- | --- | --- | --- | --- | --- | --- | --- | --- | --- | --- | --- | --- | --- |
|  |  |  |  | **OD** | **OS** | **OD** | **OS** | **OD** | **OS** | **OD** | **OS** |  |  |  |  |  |
| KC15 | KTCN | F | 46 | - | - | 164 | 303 | *22.73* | *21.72* | 7 | 11 | yes | no | no | DES, glaucoma, keratitis history | allergies |
| KC16 | KTCN | M | 30 | - | - | 303 | 314 | *24.62* | *23.74* | 13 | 13 | no | no | no | no | no |
| KC17 | KTCN | F | 46 | 204 | nd | 272 | nd | *24.20* | *22.43* | 10 | 9 | no | yes | no | no | no |
| KC18 | KTCN | M | 35 | 68 | 152 | 444 | 366 | *24.19* | *23.82* | 11 | 10 | no | no | no | no | no |
| KC19 | KTCN | M | 51 | 92 | 254 | 446 | 359 | *27.60* | *27.35* | 15 | 13 | no | yes | no | allergic conjunctivitis, history of ocular trauma | hypertension |
| KC20 | KTCN | M | 31 | nd | nd | nd | nd | *23.30* | *23.60* | nd | nd | no | yes | no | no | no |
| KR19 | non-KTCN | F | 61 | nd | nd | nd | nd | 23.35 | nd | 13 | 12 | no | - | - | descemetocoele, DES, cataract, history of corneal perforation OD and uveitis OU | rheumatoid arthritis, ulcerative colitis |
| KR21 | non-KTCN | F | 71 | nd | nd | nd | nd | 22.92 | 22.93 | 16 | 17 | no | - | - | descemetocoele, cataract, corneal ulcer | diabetes, coronary artery disease, rheumatoid arthritis, aortic insufficiency |
| KR23 | non-KTCN | F | 64 | nd | nd | nd | nd | nd | nd | 13 | 18 | no | - | - | bullous keratopathy, cataract | asthma, allergy, hypertension, psoriasis |
| KR24 | non-KTCN | M | 43 | nd | nd | nd | nd | 25.43 | 25.20 | 10 | 12 | no | - | - | keratitis, history of ocular trauma | asthma, allergy, hypertension |
| KR25 | non-KTCN | F | 78 | nd | nd | nd | nd | 22.26 | 22.19 | 13 | 14 | no | - | - | bullous keratopathy, cataract, glaucoma, history of keratitis and uveitis OU, diabetic retinopathy | hypertension, diabetes, multiple myeloma |
| KR49 | non-KTCN | M | 38 | nd | nd | nd | nd | 24.15 | 24.5 | 14 | 15 | no | - | - | history of ocular trauma | no |

Abbreviations in table: OD – right eye, OS – left eye, OU – both eye, nd – data not available, IOP - intraocular pressure, AL - axial length, DES - dry eye syndrome

*italic font in AL [mm] column indicates a measurement made before surgery
