# Supplementary material for: Further evaluation of differential expression of keratoconus candidate genes in human corneas
Source: PeerJ. 2020 Aug 20;8:e9793. doi: 10.7717/peerj.9793 (PMC7443321; doi:10.7717/peerj.9793)
Supplement: Supplemental Information 2 [file peerj-08-9793-s002.docx]

Table S2. Genes selected for RT-qPCR analysis using Real Time ready Custom Panels (Roche)

| **Gene**  **symbol** | **Protein name** | **HGNC ID** | **ENSEMBL ID** |
| --- | --- | --- | --- |
| *Genes that have been selected based on RNA-seq study from top molecular pathways overrepresented across deregulated genes, encoding core elements of collagen synthesis and maturation pathways, the TGF-β, Hippo, and Wnt signaling pathways, as well as their potential regulators* | | | |
| *TGFB1* | Transforming Growth Factor Beta 1 | 11766 | ENSG00000105329 |
| *TGFB2* | Transforming Growth Factor Beta 2 | 11768 | ENSG00000092969 |
| *TGFB3* | Transforming Growth Factor Beta 3 | 11769 | ENSG00000119699 |
| *TGFBR1* | Transforming Growth Factor Beta Receptor 1 | 11772 | ENSG00000106799 |
| *TGFBR2* | Transforming Growth Factor Beta Receptor 2 | 11773 | ENSG00000163513 |
| *CTGF* | Connective Tissue Growth Factor | 2500 | ENSG00000118523 |
| *COL5A2* | Collagen Type V Alpha 2 Chain | 2210 | ENSG00000204262 |
| *COL21A1* | Collagen Type XXI Alpha 1 Chain | 17025 | ENSG00000124749 |
| *LOX* | Lysyl Oxidase | 6664 | ENSG00000113083 |
| *BMP1* | Bone Morphogenetic Protein 1 | 1067 | ENSG00000168487 |
| *BMP4* | Bone Morphogenetic Protein 4 | 1071 | ENSG00000125378 |
| *ZFYVE9* | Zinc Finger FYVE-Type Containing 9 | 6775 | ENSG00000157077 |
| *PPP2R2B* | Protein Phosphatase 2 Regulatory Subunit Bbeta | 9305 | ENSG00000156475 |
| *WNT5A* | Wnt Family Member 5A | 12784 | ENSG00000114251 |
| *SMAD7* | SMAD Family Member 7 | 6773 | ENSG00000101665 |
| *SMAD9* | SMAD Family Member 9 | 6774 | ENSG00000120693 |
| *ACTB* | Actin Beta | 132 | ENSG00000075624 |
| *TEAD2* | TEA Domain Transcription Factor 2 | 11715 | ENSG00000074219 |
| *TEAD3* | TEA Domain Transcription Factor 3 | 11716 | ENSG00000007866 |
| *TEAD4* | TEA Domain Transcription Factor 4 | 11717 | ENSG00000197905 |
| *DNMT1* | DNA Methyltransferase 1 | 2976 | ENSG00000130816 |
| *DNMT3A* | DNA Methyltransferase 3 Alpha | 2978 | ENSG00000119772 |
| *DNMT3B* | DNA Methyltransferase 3 Beta | 2979 | ENSG00000088305 |
| *EZH2* | Enhancer Of Zeste 2 Polycomb Repressive Complex 2 Subunit | 3527 | ENSG00000106462 |
| *YY1* | YY1 Transcription Factor | 12856 | ENSG00000100811 |
| *YY1AP1* | YY1 Associated Protein 1 | 30935 | ENSG00000163374 |
| *Genes that have been previously reported as involved in KTCN based on their function, detection of putative variants within the gene, localization within the linkage region, or localized in proximity to variants associated with KTCN* | | | |
| *ZNF469* | Zinc Finger Protein 469 | 23216 | ENSG00000225614 |
| *DOCK9* | Dedicator Of Cytokinesis 9 | 14132 | ENSG00000088387 |
| *HGF* | Hepatocyte Growth Factor | 4893 | ENSG00000019991 |
| *IL1RN* | Interleukin 1 Receptor Antagonist | 6000 | ENSG00000136689 |
| *SKP1* | S-Phase Kinase-Associated Protein 1 | 10899 | ENSG00000113558 |
| *PROB1* | Proline Rich Basic Protein 1 | 41906 | ENSG00000228672 |
| *IL6* | Interleukin 6 | 6018 | ENSG00000136244 |
| *IL17B* | Interleukin 17B | 5982 | ENSG00000127743 |
| *PLEKHA7* | Pleckstrin Homology Domain Containing A7 | 27049 | ENSG00000166689 |
| *TGFBI* | Transforming Growth Factor Beta Induced | 11771 | ENSG00000120708 |
| *SPARC* | Secreted Protein Acidic And Cysteine Rich | 11219 | ENSG00000113140 |
| *FGF9* | Fibroblast Growth Factor 9 | 3687 | ENSG00000102678 |
| *FGF14* | Fibroblast Growth Factor 14 | 3671 | ENSG00000102466 |
| *ZEB1* | Zinc Finger E-Box Binding Homeobox 1 | 11642 | ENSG00000148516 |
| *SLC4A11* | Solute Carrier Family 4 Member 11 | 16438 | ENSG00000088836 |
| *WDR33* | WD Repeat Domain 33 | 25651 | ENSG00000136709 |
| *MORC1* | MORC Family CW-Type Zinc Finger 1 | 7198 | ENSG00000114487 |
| *SYN2* | Synapsin II | 11495 | ENSG00000157152 |
| *CTNNB1* | Catenin Beta 1 | 2514 | ENSG00000168036 |
| *Reference genes* | | | |
| *GAPDH* | Glyceraldehyde-3-Phosphate Dehydrogenase | 4141 | ENSG00000111640 |
| *IPO8* | Importin 8 | 9853 | ENSG00000133704 |
