# Supplementary material for: Further evaluation of differential expression of keratoconus candidate genes in human corneas
Source: PeerJ. 2020 Aug 20;8:e9793. doi: 10.7717/peerj.9793 (PMC7443321; doi:10.7717/peerj.9793)
Supplement: Supplemental Information 3 — Mean expression (mean) and standard deviations (sd) are shown. [file peerj-08-9793-s003.docx]

Table S3. Relative changes in gene expression level (rsd %) )in the KTCN corneas (KC) compared with the non-KTCN corneas (KR). Mean expression (mean) and standard deviations (sd) are shown.

| **Gene** | **KC_mean** | **KC_sd** | **KC_rsd %** | **KR_mean** | **KR_sd** | **KR_rsd %** |
| --- | --- | --- | --- | --- | --- | --- |
| *TGFB1* | 0.49 | 0.24 | 49.46 | 1.09 | 0.52 | 47.38 |
| *TGFB2* | 0.73 | 0.51 | 69.56 | 1.29 | 1.17 | 91.13 |
| *TGFB3* | 0.19 | 0.11 | 60.24 | 1.13 | 0.52 | 46.09 |
| *TGFBR1* | 0.78 | 0.14 | 18.27 | 1.03 | 0.28 | 27.29 |
| *TGFBR2* | 0.51 | 0.14 | 26.72 | 1.06 | 0.41 | 38.90 |
| *CTGF* | 0.08 | 0.09 | 106.24 | 1.49 | 1.08 | 72.01 |
| *COL5A2* | 0.22 | 0.07 | 29.45 | 1.22 | 0.91 | 74.70 |
| *COL21A1* | 14.92 | 4.89 | 32.78 | 9.01 | 20.77 | 230.45 |
| *LOX* | 0.36 | 0.18 | 51.22 | 1.38 | 1.30 | 94.78 |
| *BMP1* | 0.57 | 0.09 | 15.34 | 1.08 | 0.43 | 39.59 |
| *ZFYVE9* | 1.59 | 0.39 | 24.75 | 1.14 | 0.71 | 62.77 |
| *WNT5A* | 1.02 | 0.19 | 18.89 | 1.13 | 0.52 | 46.24 |
| *TEAD2* | 0.31 | 0.18 | 59.28 | 1.17 | 0.71 | 60.90 |
| *TEAD3* | 1.26 | 0.46 | 36.10 | 1.01 | 0.12 | 12.01 |
| *TEAD4* | 0.39 | 0.12 | 30.34 | 1.08 | 0.48 | 43.88 |
| *ZNF469* | 0.10 | 0.03 | 33.44 | 1.08 | 0.54 | 49.55 |
| *DOCK9* | 1.51 | 0.33 | 21.76 | 1.15 | 0.83 | 71.66 |
| *HGF* | 1.20 | 0.89 | 74.32 | 1.38 | 1.45 | 104.87 |
| *IL1RN* | 0.82 | 0.19 | 22.52 | 1.04 | 0.29 | 27.80 |
| *SKP1* | 1.71 | 0.34 | 19.76 | 1.05 | 0.36 | 34.81 |
| *PROB1* | 0.75 | 0.31 | 40.87 | 1.19 | 0.64 | 53.25 |
| *DNMT1* | 0.78 | 0.10 | 12.64 | 1.10 | 0.63 | 56.95 |
| *DNMT3A* | 1.36 | 0.30 | 21.84 | 1.05 | 0.32 | 30.86 |
| *DNMT3B* | 0.68 | 0.28 | 42.05 | 1.24 | 0.73 | 58.56 |
| *SMAD7* | 0.23 | 0.12 | 49.49 | 1.08 | 0.42 | 38.53 |
| *TGFBI* | 3.58 | 1.36 | 38.05 | 1.44 | 1.61 | 111.58 |
| *SPARC* | 0.28 | 0.08 | 28.97 | 1.23 | 0.89 | 72.11 |
| *EZH2* | 0.76 | 0.25 | 33.43 | 1.02 | 0.22 | 21.20 |
| *YY1* | 1.21 | 0.21 | 16.95 | 1.11 | 0.61 | 55.29 |
| *FGF9* | 3.13 | 2.11 | 67.55 | 2.04 | 2.92 | 142.86 |
| *ZEB1* | 0.60 | 0.41 | 68.46 | 1.27 | 1.17 | 91.96 |
| *SLC4A11* | 3.05 | 1.72 | 56.31 | 1.27 | 0.88 | 69.21 |
| *WDR33* | 1.39 | 0.37 | 26.90 | 1.06 | 0.42 | 39.68 |
| *CTNNB1* | 1.02 | 0.14 | 13.28 | 1.02 | 0.23 | 22.81 |
| *YY1AP1* | 1.25 | 0.14 | 11.50 | 1.05 | 0.40 | 38.23 |
| *ACTB* | 0.69 | 0.16 | 23.67 | 1.07 | 0.36 | 33.94 |
